# Thesaurus-based disambiguation of gene symbols

**DOI:** 10.1186/1471-2105-6-149

**Published:** 2005-06-16

**Authors:** Bob JA Schijvenaars, Barend Mons, Marc Weeber, Martijn J Schuemie, Erik M van Mulligen, Hester M Wain, Jan A Kors

**Affiliations:** 1 Department of Medical Informatics, Erasmus University Medical Center Rotterdam, P.O. Box 1738, 3000 DR Rotterdam, The Netherlands; 2 HUGO Gene Nomenclature Committee, Department of Biology, University College London, Wolfson House, 4 Stephenson Way, London NW1 2HE, UK

## Abstract

**Background:**

Massive text mining of the biological literature holds great promise of relating disparate information and discovering new knowledge. However, disambiguation of gene symbols is a major bottleneck.

**Results:**

We developed a simple thesaurus-based disambiguation algorithm that can operate with very little training data. The thesaurus comprises the information from five human genetic databases and MeSH. The extent of the homonym problem for human gene symbols is shown to be substantial (33% of the genes in our combined thesaurus had one or more ambiguous symbols), not only because one symbol can refer to multiple genes, but also because a gene symbol can have many non-gene meanings. A test set of 52,529 Medline abstracts, containing 690 ambiguous human gene symbols taken from OMIM, was automatically generated. Overall accuracy of the disambiguation algorithm was up to 92.7% on the test set.

**Conclusion:**

The ambiguity of human gene symbols is substantial, not only because one symbol may denote multiple genes but particularly because many symbols have other, non-gene meanings. The proposed disambiguation approach resolves most ambiguities in our test set with high accuracy, including the important gene/not a gene decisions. The algorithm is fast and scalable, enabling gene-symbol disambiguation in massive text mining applications.

## Background

The amount of information in the life sciences is staggering and growing exponentially. One of the largest biomedical resources of textual scientific information, the Medline database, currently contains over 14 million abstracts, with an estimated increase in size of more than one article per minute. Scientists are faced with an overload of information, which is particularly pressing in the biological field where high-throughput experiments in genomics and proteomics generate new data at an unprecedented rate. More often than not, interpretation of these data requires the digestion and integration of information contained in many thousands of articles and other information sources, a daunting task clearly beyond the capacity of human reading and comprehension.

Recently, a number of information retrieval systems have been proposed to extract and relate pertinent biological information from large corpora of text [1-9]. These systems even hold promise for the discovery of new, "tacit" knowledge that is hidden in the literature. The term "conceptual biology" has already been coined to distinguish this emerging field of research as a branch of biological research in its own right [10]. There are however several issues that limit the practical utility of current text-mining tools [11]. One problem is the highly-variable use of gene nomenclature in the literature [12,13], producing multiple symbols and names for one and the same gene. This complicates relating information in different documents that deal with the same gene but use different symbols. One approach to deal with this synonym problem is to make use of the information about genes and their aliases that is available in existing genetic databases.

A second, probably more intricate, problem is that a single gene symbol may refer to multiple genes, or may also be the abbreviation of terms with completely different, non-gene meanings. When building gene networks from the literature [1], for example, one would not want to contaminate the network on prostate specific antigen (*PSA*) with puromycin-sensitive aminopeptidase, psoriatric arthritis, pig serum albumin, or one of the more than 100 other meanings of PSA that can be found in the literature [14].

The extent of this ambiguity or homonym problem has been further subject of two recent studies. Tuason *et al. *[15] compared gene symbols of four organisms (not including human) and showed that up to 20% of the gene symbols of an individual organism were ambiguous with the other three organisms. In another study by the same group, Chen *et al. *[16] found that 85% of correctly retrieved mouse genes in a set of 45,000 abstracts were ambiguous with gene names from 20 other organisms, while ignoring gene names that were also English words. When the latter were included, 233% additional "gene" instances were retrieved, most of which were false positives. In several other studies [17-19], it was also suggested that solving this ambiguity problem is an important requirement for large-scale application of text-mining tools in the biomedical field.

General word-sense disambiguation has been studied extensively in the field of natural language processing. A wide variety of approaches has been proposed (see [20,21] for excellent reviews), including dictionary-based approaches and the use of supervised learning techniques to build classifiers that assign the proper sense to an ambiguous term. Typically, these methods use the words in a window around the ambiguous term, or information derived from this context window, such as part-of-speech or collocation.

Recently, several studies have explored the use of disambiguation techniques in the biological field. Hatzivassiloglou *et al. *[22] applied machine learning methods to classify symbols into one of three categories: genes, proteins, and mRNA. No attempt was made to resolve homonyms with two or more senses within one group, or with a sense outside of these three groups, and performance results were rather moderate, although still better than human interpretation. The same problem was recently tackled by Ginter *et al. *[23], who proposed a new classifier design and were able to slightly improve on the best method used by Hatzivassiloglou [22]. In a series of articles [17,24,25], Liu and co-workers investigated the effect of different supervised learning techniques, feature representations, and context window sizes on disambiguation performance. They obtained excellent results on a small number of ambiguous biomedical abbreviations [17,24], but for training they typically needed dozens of examples for each of the possible senses. In practice, these numbers may often be difficult to obtain. Widdows *et al. *[26] compared several methods for disambiguating ambiguous concepts from the Medical Subject Headings (MeSH) thesaurus [27] on a set of 70 ambiguous terms. Their most successful method achieved 74% precision and utilized existing MeSH-term co-occurrence data, which were derived from the MeSH annotations by human annotators. However, their method would not work well for gene symbols, which are poorly covered by MeSH.

Recently, Podowski *et al. *[19] used Bayesian classifier models to disambiguate gene symbols found in LocusLink [28]. Interestingly, their system can distinguish between gene and non-gene meanings of a symbol, acknowledging the fact that many gene symbols are abbreviations of terms with non-gene meanings. They validated their system on two manually curated test sets of 66 gene symbols, and found that the accuracy of the system is mostly over 90% when more than 20 abstracts per gene sense were available for training.

Although several of these approaches produced very good disambiguation results, they require substantial amounts of training data, typically tens of instances per sense. For gene symbol disambiguation, these numbers may be difficult to acquire. Given the extent of the homonym problem for gene symbols, manual curation of training data would be extremely laborious. Any practical disambiguation system should be trained with data that are gathered automatically, but even then the required numbers are unlikely to be available for many of the ambiguous symbols.

Here we present a disambiguation method for gene symbols, which maintains excellent performance when trained with sparse data. At the basis of our approach lies a thesaurus that is used to find biomedical concepts, including gene symbols, in text. Focusing on human genes, we first quantify the ambiguity problem for gene symbols, particularly paying attention to ambiguity arising from non-gene meanings of gene symbols. We then describe our disambiguation approach and assess the performance of the disambiguation algorithm on a large test set of documents.

## Results

### Thesaurus construction and ambiguity of human gene symbols

We extracted human gene symbols and aliases, gene names, and identification numbers from five publicly available databases [29]: Genew, the Genome Database (GDB), LocusLink, Online Mendelian Inheritance in Man (OMIM), and Swiss-Prot. Genes from the different databases were matched based on identification numbers and overlap in gene symbols or names, and the corresponding information was merged into a new gene thesaurus. The resulting thesaurus contained information on 26,367 human genes, with a total of 63,148 gene symbols. The percentage of ambiguous gene symbols was 4.9% (2,911 of 59,604 distinct gene symbols), whereas the percentage of genes affected by homonymy was 17.5% (4,606/26,367).

**Figure 1:**
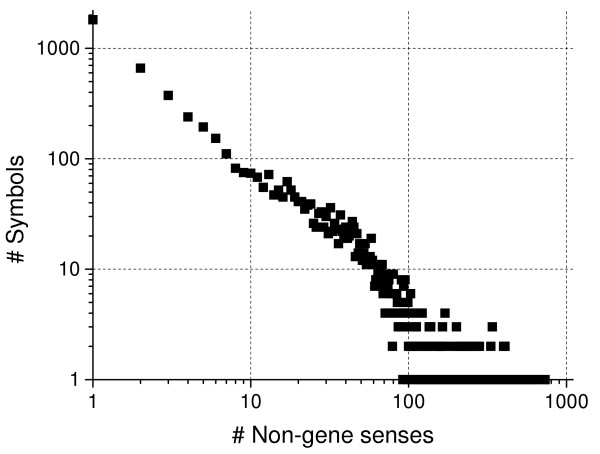
**Number of non-gene meanings for gene symbols. **Dots indicate the number of human gene symbols (on the vertical axis) and, for each of these symbols, the number of corresponding long forms with a non-gene meaning (horizontal axis). It should be noted that spelling variations may yield different long forms for the same non-gene meaning. To reduce the effect of these variations, long forms were stemmed.

Gene symbols may not only denote multiple genes, but may also have other, non-gene meanings. To further gauge the extent of ambiguity, we searched 13 years of Medline abstracts for abbreviations (or short forms) and their expansions (or long forms) using an abbreviation expansion algorithm [30]. A total of 10,398 unique, case-sensitive short forms were found that matched a gene symbol from our gene thesaurus, with 146,198 long forms. Of these, 117,149 long forms (corresponding with 5,639 symbols) did not match, partially or completely, any of the gene names associated with that symbol, and were assumed to have a non-gene meaning. The number of different long forms per symbol with a non-gene meaning varies widely (Figure 1), up to 734 (for the short form "PC", which for example can denote "pachyonychia congenital", "prefrontal cortex", and "protective clothing"). The short forms with at least one non-gene meaning affected 26.9% of the genes and 9.5% of the gene symbols in our combined gene thesaurus. Overall, taking into account both gene and non-gene meanings, 32.7% of the genes in our combined thesaurus had one or more homonymous symbols, and 12.6% of the gene symbols in the thesaurus were ambiguous.

### Disambiguation of gene symbol senses

The algorithm to disambiguate homonymous gene symbols operates as follows. For each of the possible genes that the symbol can denote, a reference description is assumed to be available. Given an ambiguous symbol, the textual context in which it occurs, say, a Medline abstract, is matched with each reference description, yielding a set of matching scores. The gene corresponding with the reference description that best matches the context is then taken to indicate the symbol's meaning. However, the symbol is assumed to have a non-gene meaning if the context does not match well with any of the reference descriptions and the matching score stays below a homonym-dependent threshold, as determined by a leave-one-out procedure (see Additional file: 1 for an example of the disambiguation process). For the textual context, we used the title, abstract, and MeSH terms that had been assigned to the Medline abstract. As reference descriptions, we selected either gene annotations that were culled from OMIM, or one or more (up to five) Medline abstracts about a particular gene.

For training and testing purposes, we automatically compiled a set of annotations and abstracts for 690 ambiguous symbols, having 974 different gene meanings; 528 of the symbols had at least one non-gene meaning. All abstracts and annotations were sought for concepts from MeSH and the gene thesaurus with indexing software from Collexis (Geldermalsen, The Netherlands) [31]. For each document this yielded a list of biomedical concepts with attached relevance scores (a "concept fingerprint", or CFP), which was used for subsequent processing.

For each gene sense of a symbol, five randomly chosen abstracts were set aside for generating different reference CFPs; the remaining abstracts were used for testing. The test set contained 52,529 abstracts. The matching score between textual context and reference description was defined as the normalized cosine-vector score [32] between the CFPs of these two texts.

**Figure 2:**
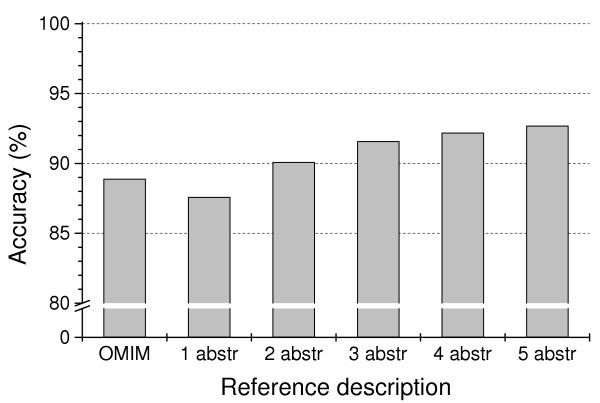
**Performance of the disambiguation algorithm. **Total accuracies of the disambiguation algorithm were determined on the test set of 52,529 Medline abstracts for reference fingerprints derived from different reference descriptions: OMIM annotations or a varying number of Medline abstracts. When two or more abstracts were used, the fingerprints of the individual abstracts were averaged to yield the final reference fingerprint.

Overall accuracy of the disambiguation algorithm, defined as the percentage of abstracts in our test set in which the correct meaning of the homonym was chosen, was 88.9% when OMIM annotations were used as reference description. This was comparable to using one abstract as the reference (87.6%), while accuracy increased to 92.7% when a CFP combination of five abstracts was used as the reference (Figure 2). For comparison, a simple majority rule (for each symbol always select the sense that occurs most often in the test set) resulted in a baseline accuracy of 72.4%.

We made a breakdown of the errors when a combination of five abstracts was used as the reference description. As shown in Table 1, symbols indicating a gene were assigned a non-gene meaning in 6.5% of the cases; symbols with a non-gene meaning were misclassified as a gene even less frequently (4.5%). For gene symbols with multiple gene meanings, 9.9% of the symbols were assigned to the incorrect genes (993 out of 10,054 abstracts that contain an in-thesaurus homonym).

**Table 1:** Disambiguation of gene vs. non-gene senses. Table entries show the number of abstracts in the test set with gene symbols that were correctly or incorrectly classified by the disambiguation algorithm as having a gene or non-gene sense. The percentages indicate the correctly and incorrectly classified symbols relative to the row totals. Reference fingerprints per gene symbol sense were derived from a combination of five Medline abstracts, not being part of the test set.

	Algorithm	
	
Reference	Gene	Non-gene
Gene	24243 (93.5%)	1666 (6.5%)
Non-gene	1197 (4.5%)	25323 (95.5%)

## Discussion

Ambiguity of gene symbols in free text is an impediment for the massive application of text mining and literature-based discovery methods. Our assessment of gene symbol ambiguity indicates that the homonym problem cannot be ignored when text mining in the biological field is performed, corroborating findings of previous studies [15]. We found up to 33% of the genes in our thesaurus being affected by homonymy, and even this high figure is underestimating the problem because we limited ourselves to human genes only, not considering other organisms and gene products.

Disambiguation would be a trivial task if each ambiguous symbol in an abstract were accompanied by its corresponding long form at least once. Unfortunately, this approach is of limited practical value. We recently checked 3,901 Nature Genetics and BioMed Central articles and found that only 30% of the gene symbols in the abstracts are accompanied by a matching long form [33]. For an additional 8% of the symbols in the abstracts, the long form could be found in the full text. Of all gene symbols mentioned in the full-text articles, only 18% were accompanied by a long form.

We compared two sources of reference descriptions, gene annotations and abstracts about a particular gene. OMIM annotations did not perform better as a reference description than randomly chosen abstracts about a gene. The performance increased when the reference CFP was constructed from the information of several abstracts combined, but the improvement appeared marginal when more than three abstracts were used (Figure 2). This suggests that excellent disambiguation results can be obtained with relatively simple reference descriptions, and offers a viable way for massive acquisition of such descriptions from literature links in genetic databases, which in view of the extent of the homonym problem should be automatic for all practical purposes.

Our disambiguation algorithm could be used as part of a gene identification module in an information extraction application. In a recent review article on term identification, Krauthammer and Nenadic [34] distinguish between two types of disambiguation: at the broader level of pinpointing the type of a concept (e.g., distinguishing between genes and non-genes) and at the specific level of resolving different meanings of a term within a term class (e.g., distinguishing between homonymous genes within a gene thesaurus). Our approach addresses both types of disambiguation. While the algorithm was developed and tested for disambiguation of gene symbols, the approach is general and easy to apply to other ambiguous entities as well, provided adequate reference descriptions are available.

We would like to emphasize the practicality of our approach in terms of processing speed, scalability, and accuracy. The initial indexing process is the most time-consuming step, but in principle has to be done only once. For our whole test set of 52,529 abstracts, indexing currently takes about two hours on a standard Pentium IV computer. Once the context and reference fingerprints are available, the disambiguation process itself is very fast, taking about two minutes for the whole test set.

In practical applications, reference descriptions will be needed for many more than the almost 700 homonymous gene symbols that we used in this study. Considering that even a single abstract about a particular gene can provide an adequate reference description, our approach can easily be scaled up, for instance by taking the abstracts that are referenced with each of the gene descriptions in LocusLink. In our download of LocusLink, 13,811 genes had at least one reference, and 7,587 had three or more.

Automatic determination of a gene/non-gene threshold may be more difficult, as our approach presently requires the availability of examples of non-gene meanings of a symbol. We are currently investigating automatic threshold setting based on a general set of abstracts with non-gene meanings, obviating the need to acquire non-gene examples of each specific symbol.

The accuracy of any disambiguation algorithm must be very high in order to be of practical value in massive literature mining. In this respect, gene symbols that are assigned a non-gene meaning (6.5% in our test set) may be less of a problem than the other way around (4.5%), or than being assigned the wrong gene meaning (9.9%). It should be remarked that these results pertain to our test set, which is large but still limited in scope because we only selected gene symbols that occur in OMIM and had six or more abstracts per gene sense. This may have favored selection of relatively well-known genes. It is conceivable that some abstracts with gene symbols that were not selected provide a less focussed context that would perform less well.

In our data selection, we focused on human genes. The abstracts in our test set with ambiguous symbols that indicated a gene, were taken from two sources: OMIM, which is a database about human genes and diseases, or the SF/LF data set, if both short form and long form in the abstract exactly matched an entry in our human gene thesaurus. However, previous investigations [15,16] showed that substantial ambiguity of gene symbols exists across species, and suggested that most of this ambiguity was attributable to homologous genes. In a random sample of 100 abstracts from our test set with symbols that had a gene meaning, 31 symbols referred to non-human genes, mostly from mouse or rat (data not shown). All of these were apparently homologous to the human genes with identical names. In this study, we did not attempt to distinguish between homologous genes. Model organisms are often used to understand the biology of human genes and the distinction between homologous genes in text is often difficult to make, or not useful. If disambiguation of homologous genes is important, though, our approach could be extended by including reference descriptions of genes from other species.

Finally, the current performance is based on reference descriptions that were acquired fully automatically. Manual curation of these descriptions or their corresponding fingerprints for low-scoring symbols may further add to the algorithm's performance.

## Conclusion

The ambiguity of gene symbols is substantial, not only because one symbol may denote multiple genes but particularly because many symbols have other, non-gene meanings. A simple, thesaurus-based disambiguation approach can resolve most ambiguities in our test set with high accuracy, including the important gene/not a gene decisions. The proposed method is fast and scalable, enabling gene-symbol disambiguation in massive text mining and information extraction applications.

## Methods

### Construction of the gene thesaurus

We downloaded (January 2004) information about human genes from five curated databases: Genew [35], GDB [36], LocusLink [37], OMIM [38], and Swiss-Prot [39]. For each human gene in a database, gene symbols (including aliases), gene names, and gene identification codes were extracted. Since gene name fields in the databases often contain more descriptive statements rather than gene names proper, we excluded gene names that could not be matched with one of the corresponding gene symbols according to the abbreviation expansion algorithm described by Schwartz and Hearst [30].

The number of identification codes per gene varied from database to database. Each database maintains its own set of gene identification codes, but also included cross-references to one or more of the other databases; also gene identification codes from Unigene and RefSeq were extracted if available. The original databases, including Unigene and RefSeq, were searched for information about obsolete identification codes and their possible replacements, and the extracted codes were corrected or excluded as appropriate.

To find corresponding genes from the different databases, genes with any matching identification code, gene symbol, or gene name were grouped. Within each group, subgroups of genes without conflicting identification codes were generated. If there was only one subgroup, the genes in this subgroup were taken to represent one and the same gene and all gene symbols and names of the separate genes were merged. If there was more than one subgroup, an iterative procedure was entered in which the number of similar and disparate identification codes as well as the overlap in gene symbols and names were determined for all bigroup comparisons of subgroups. A scoring rule was then used to decide whether two subgroups represented the same gene and should be merged.

The new gene thesaurus contained information on 26,367 human genes, with a total of 63,148 gene symbols. The overlap with the original databases is most substantial for LocusLink, which covers 98.1% of the genes and 92.3% of the gene symbols in the new thesaurus; Genew, maintained by the HUGO Gene Nomenclature Committee, covers 67.0% of the genes and 54.3% of the symbols. The average number of symbols per gene in the original databases varies from 1.68 (in OMIM) to 2.25 (in LocusLink); the combined gene thesaurus has an average of 2.39 symbols per gene.

### Text indexing

Text documents were indexed with Collexis (Geldermalsen, The Netherlands) indexing software. For a given text, frequently occurring non-informative words are removed and the remaining terms are stemmed (using the LVG software which is part of the UMLS lexical tools [40]), i.e., brought into a standard, canonical form.

Subsequently, the document is searched for biomedical terms that occur in MeSH or in our gene thesaurus. Each found term is mapped to a unique identification code that denotes the preferred term, or concept, *t*_*i *_and is assigned a relevance score or weight *w*_*i *_that equals the Term Frequency *TF *(the number of occurrences *f*_*i *_of the concept *t*_*i *_(i.e., the term or its synonyms) in the document) multiplied by the Inverse Document Frequency *IDF *(a correction factor for the number of documents *N*_*i *_containing *t*_*i *_in a given set of *N *documents; we used 10 years of Medline) [32]. We applied a commonly-used variant of the *IDF *that normalizes for the total number of documents [41]:



A document is then represented by an *M*-dimensional vector *W *= (*w*_*1*_,*w*_*2*_,...,*w*_*M*_), where *M *is the number of distinct concepts in the thesaurus, and *w*_*i *_= 0 if *t*_*i *_is not in the document. This weight vector *W *will subsequently be called the "concept fingerprint" (CFP) of the document, and is used for subsequent processing.

### Document test sets

**Figure 3:**
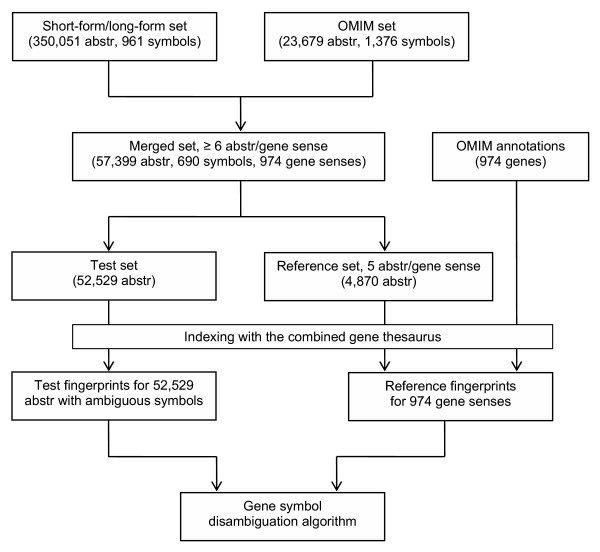
**Steps involved in the construction of test and reference fingerprints. **Two sets of abstracts containing symbols with known gene or non-gene meaning were constructed. One set consisted of abstracts with short-form/long-form combinations culled from Medline, the other set consisted of abstracts that were mentioned in OMIM annotations of genes. The two sets were merged by selecting symbols that occurred in both sets and had at least six abstracts for each of their gene senses. The OMIM annotations for the genes in the merged set were stored separately. A reference set was generated by randomly selecting five abstracts per gene sense from the merged set; the remaining abstracts were used for testing. All abstracts in the test and reference set as well as the OMIM annotations were indexed using the combined gene thesaurus, and the resulting "concept fingerprints" were used for reference fingerprint construction and testing of the disambiguation algorithm.

The various processing steps related to the construction of our test sets are summarized in Figure 3 and will be described below. We automatically generated two sets of abstracts containing symbols with known meaning. First, we searched all abstracts from Medline 1990–2002 for abbreviations, or short forms, and their corresponding expansions, or long forms, with the abbreviation expansion algorithm of Schwartz and Hearst [30]. For each homonymous short form, i.e., a short form with at least two different long forms, the gene thesaurus was mined for genes with a symbol and name that fully matched one of the short form/long form pairs. The abstracts in which these pairs occurred were labeled as containing the gene. If the long form only partially matched the names of a particular gene, we excluded the short form/long form pair and related abstracts from further consideration in order to guard against a non-gene meaning being assigned to a gene, or a gene meaning to an incorrect other gene. The remaining pairs, which could not be matched against the gene thesaurus, were lumped together in a non-gene meaning of the short form, and the corresponding abstracts were labeled accordingly. Thus, a test set of abstracts was collected containing short forms that represent either gene symbols or other meanings, excluding abstracts with uncertain meanings (short form/long form test set, 350,501 abstracts for 961 homonymous gene symbols) [14]. This approach to automatically create a sense-tagged set of abbreviations was originally proposed by [17], although they did not match against a thesaurus to focus on gene symbols and non-gene meanings.

Second, we extracted from our gene thesaurus all homonymous genes that had an OMIM identification code and a symbol that referred to multiple genes in the thesaurus or to a long form with non-gene meaning. For each gene, we culled the corresponding annotation from the OMIM database, including the PubMed citations that were given as a reference for the gene. It should be noted that if the Medline abstract contained a synonym of the homonymous gene symbol, we replaced the synonym with the homonymous symbol. All these abstracts together formed a second test set (OMIM test set, 23,678 abstracts for 1,376 gene symbols). The short form/long form set is much larger than the OMIM set because it contains many abstracts with symbols that have non-gene meanings.

In order to compare the performance of the disambiguation algorithm when using either annotations or abstracts as the reference descriptions (see below), symbols were selected that occurred in both our test sets and had at least six abstracts for each of its gene senses. A total of 690 symbols qualified, having 974 different gene meanings; 528 of the symbols had at least one non-gene meaning. For each gene sense of a symbol, five randomly chosen abstracts were set aside for generating a reference CFP; the remaining abstracts, with a maximum of 100 abstracts per sense, were used for testing. The test set contained 52,529 abstracts, 25,809 with symbols having a gene meaning and 26,720 with symbols having a non-gene meaning.

### Homonym disambiguation

The disambiguation algorithm compares the textual context of a homonym in a document with a reference description of each of the genes that the homonym may possibly denote. For the textual context, we took the CFP of the abstract (including title and MeSH terms) in which the homonym occurs, setting the weight of the homonym itself to 0. For the reference descriptions, two approaches were studied. In one approach, the CFP of the OMIM annotation of a gene served as the reference. In the other approach, an averaged reference CFP was derived from the CFPs of abstracts (up to five) with a known sense of the homonym, by summing the term frequencies of the individual abstracts and computing the weights. In either case, the context CFP *W*_*c *_and the reference CFP *W*_*r *_were then compared by computing a normalized cosine-vector score [32]:



where *w*_*ci *_and *w*_*ri *_are the relevance scores of concept *t*_*i *_in the context CFP and the reference CFP, respectively, and |*W*_*c*_| and |*W*_*r*_| are the lengths of these CFPs. The cosine score varies between 1 (identical CFPs) and 0 (no overlap between CFPs). For each gene sense of the homonym, a score was determined and the sense with the highest score was assigned to the term, unless the maximum score was lower than a homonym-dependent threshold value, in which case the homonym was taken to have a non-gene meaning. Error rates were determined with a leave-one-out procedure: for each of the test abstracts of a particular gene symbol (containing both gene and non-gene meanings), scores between the context CFP and each of the reference CFPs for that symbol were determined and the highest score selected. (If there was only one reference CFP for the symbol, there was obviously only one score per abstract, which was selected.). From this set of scores, one score was left out and the threshold that minimized the error rate on the remaining scores was determined. The symbol in the abstract associated with the removed score was then classified as having a gene or non-gene meaning, depending on whether the removed score was higher or lower than this threshold value. This was done for each score in turn, yielding an overall error rate for the ambiguous symbol. The threshold that minimized the error rate for the whole sample of scores was then taken as the final homonym-dependent threshold.

## Authors' contributions

BJAS designed the study, developed and implemented the disambiguation algorithm and performed most of the data analysis. BM participated in the design of the study and helped to draft the manuscript. MW participated in building the gene thesaurus and generating the training and test data, and helped to draft the manuscript. MJS participated in the design of the study and in the data analysis. EMVM was involved in text indexing. HMW participated in the gene thesaurus construction. JAK conceived of the study, participated in its design and coordination and drafted the manuscript. All authors read and approved the final manuscript.

## Supplementary Material

Additional File 1 This file illustrates the disambiguation process by a specific example. Click here for file
