# Supplementary material for: Thesaurus-based disambiguation of gene symbols
Source: BMC Bioinformatics. 2005 Jun 16;6:149. doi: 10.1186/1471-2105-6-149 (PMC1183190; doi:10.1186/1471-2105-6-149)
Supplement: Additional File 1 — This file illustrates the disambiguation process by a specific example. [file 1471-2105-6-149-S1.pdf]

Disambiguation step-by-step

In the following, the various steps in the disambiguation process will be illustrated by an example.

Figure 1 shows a text containing the ambiguous symbol PSA. In the thesaurus that we use, PSA can indicate three different genes: “prostate-specific antigen”, “protein S (alpha)” (PROS1), and “puromycin sensitive aminopeptidase” (NPEPPS). These meanings of PSA will be called in-thesaurus meanings. However, PSA may also denote many other concepts that are not contained in our thesaurus (not-in-thesaurus meanings).

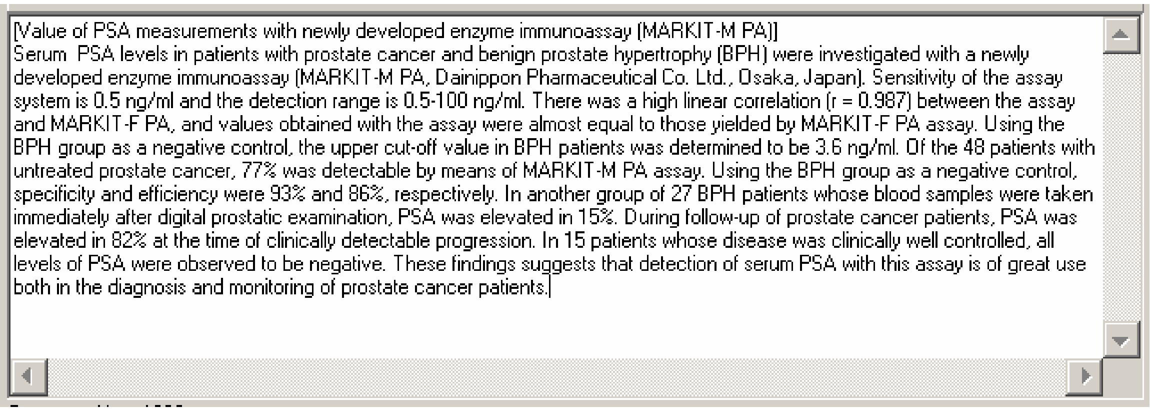
[Value of PSA measurements with newly developed enzyme immunoassay (MARKIT-M PA)]
Serum PSA levels in patients with prostate cancer and benign prostate hypertrophy (BPH) were investigated with a newly developed enzyme immunoassay (MARKIT-M PA, Dainippon Pharmaceutical Co. Ltd., Osaka, Japan). Sensitivity of the assay system is 0.5 ng/ml and the detection range is 0.5-100 ng/ml. There was a high linear correlation (r = 0.987) between the assay and MARKIT-F PA, and values obtained with the assay were almost equal to those yielded by MARKIT-F PA assay. Using the BPH group as a negative control, the upper cut-off value in BPH patients was determined to be 3.6 ng/ml. Of the 48 patients with untreated prostate cancer, 77% was detectable by means of MARKIT-M PA assay. Using the BPH group as a negative control, specificity and efficiency were 93% and 86%, respectively. In another group of 27 BPH patients whose blood samples were taken immediately after digital prostatic examination, PSA was elevated in 15%. During follow-up of prostate cancer patients, PSA was elevated in 82% at the time of clinically detectable progression. In 15 patients whose disease was clinically well controlled, all levels of PSA were observed to be negative. These findings suggests that detection of serum PSA with this assay is of great use both in the diagnosis and monitoring of prostate cancer patients.

Figure 1. Text containing the ambiguous symbol PSA.

The text is submitted to an indexing program (we used the Collexis indexing engine), which locates terms in the text that are present in the thesaurus (Figure 2). The found terms are mapped to a preferred term, or concept, and are assigned relevance scores, yielding the “concept fingerprint” of the text (Figure 3). Note that the ambiguous symbol, PSA, maps to multiple concepts.

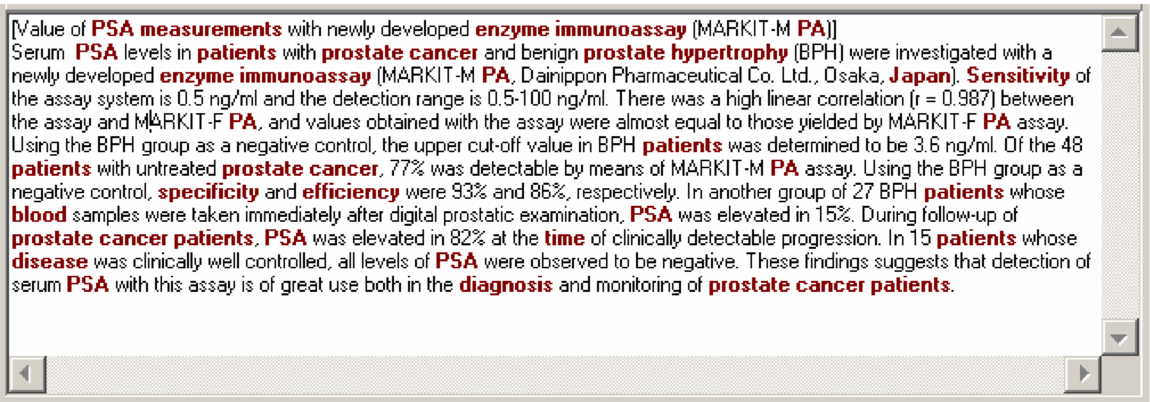
[Value of **PSA measurements** with newly developed **enzyme immunoassay** (MARKIT-M **PA**)]
Serum **PSA** levels in **patients** with **prostate cancer** and benign **prostate hypertrophy** (BPH) were investigated with a newly developed **enzyme immunoassay** (MARKIT-M **PA**, Dainippon Pharmaceutical Co. Ltd., Osaka, **Japan**). **Sensitivity** of the assay system is 0.5 ng/ml and the detection range is 0.5-100 ng/ml. There was a high linear correlation (r = 0.987) between the assay and MARKIT-F **PA**, and values obtained with the assay were almost equal to those yielded by MARKIT-F **PA** assay. Using the BPH group as a negative control, the upper cut-off value in BPH **patients** was determined to be 3.6 ng/ml. Of the 48 **patients** with untreated **prostate cancer**, 77% was detectable by means of MARKIT-M **PA** assay. Using the BPH group as a negative control, **specificity** and **efficiency** were 93% and 86%, respectively. In another group of 27 BPH **patients** whose **blood** samples were taken immediately after digital prostatic examination, **PSA** was elevated in 15%. During follow-up of **prostate cancer patients**, **PSA** was elevated in 82% at the **time** of clinically detectable progression. In 15 **patients** whose **disease** was clinically well controlled, all levels of **PSA** were observed to be negative. These findings suggests that detection of serum **PSA** with this assay is of great use both in the **diagnosis** and monitoring of **prostate cancer patients**.

Figure 2. The indexed text, with the terms contained in the thesaurus marked red.

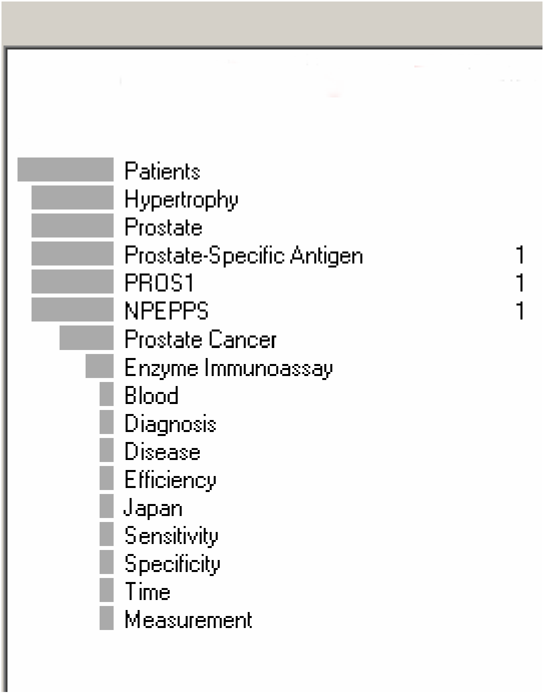

Figure 3. The concept fingerprint corresponding to the text of Figure 2. The bars on the left indicate the relevance scores attached to the concepts. The three in-thesaurus concepts that can be denoted by the term PSA, are marked by 1's.

For each of the three in-thesaurus gene senses of PSA, a reference fingerprint is available (Figures 4 to 6). Each reference fingerprint is matched with the text fingerprint (Figure 3), yielding a set of matching scores (normalized cosine-vector score). The gene corresponding to the reference fingerprint that yields the highest score is then taken to indicate the meaning of PSA in the given text. Figure 7 shows the disambiguated fingerprint.

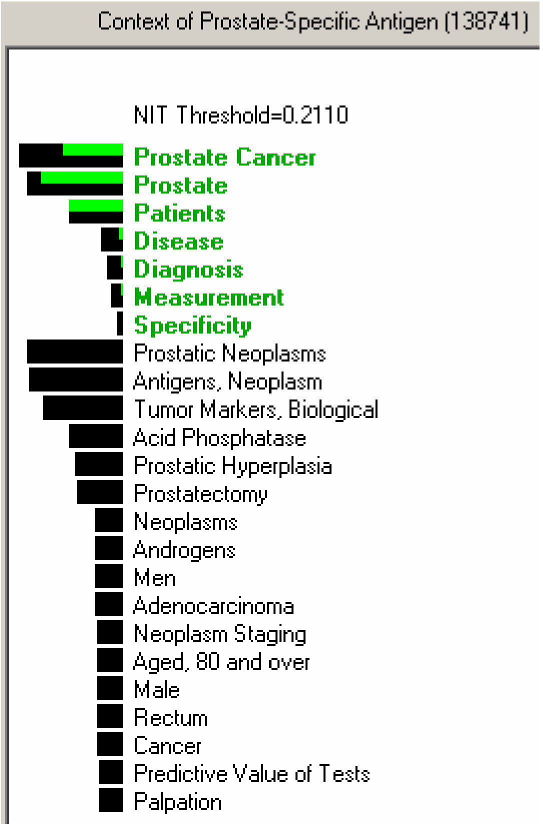

Figure 4. Reference fingerprint for the concept “Prostate-Specific Antigen”. Concepts that occur both in the reference fingerprint and in the fingerprint of the text are marked green. The green bars indicate the relative amount of overlap.

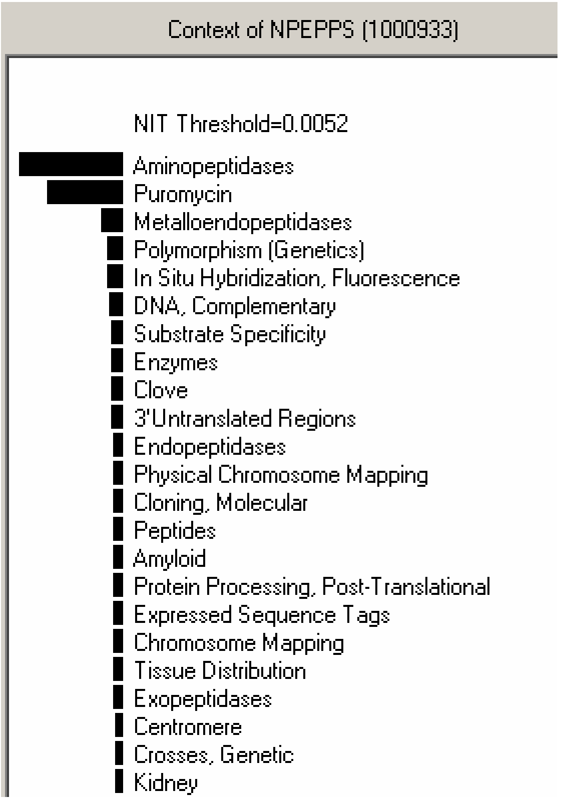

Figure 5. Reference fingerprint for the concept “NPEPPS”. Note that there are no overlapping concepts with the text fingerprint.

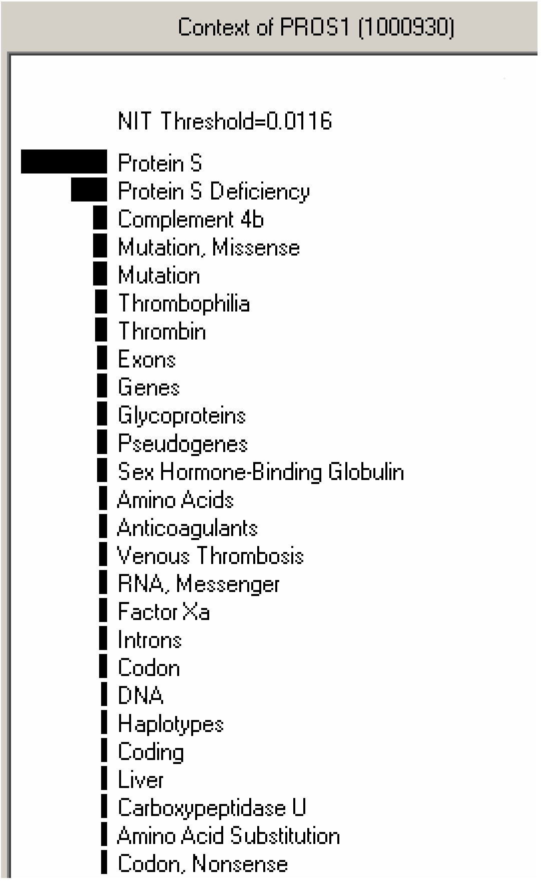

Figure 6. Reference fingerprint for the concept “PROS1”.

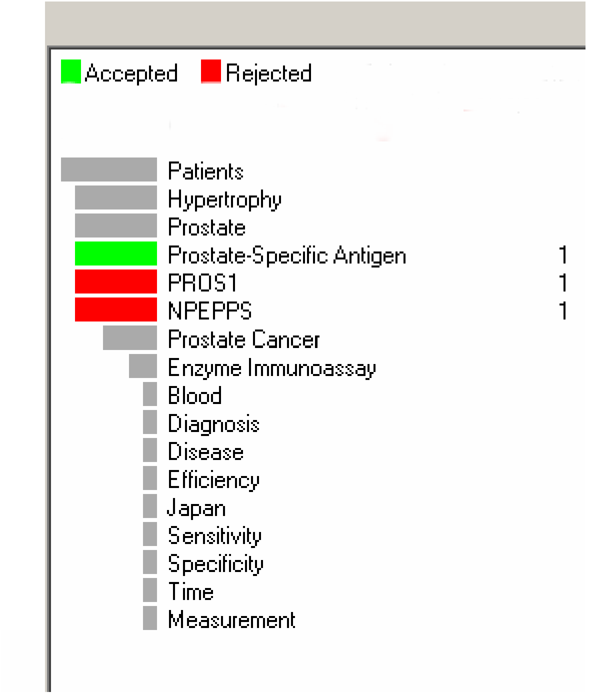

Figure 7. Disambiguated text fingerprint. The disambiguation algorithm selected the concept “Prostate-Specific Antigen”, which in the given text is the correct meaning for PSA.

However, if the maximum matching score is lower than the not-in-thesaurus threshold, the symbol is considered to have a not-in-thesaurus meaning. Figure 8 shows an example of an indexed text with a not-in-thesaurus meaning of PSA (porcine serum albumin). PSA again is recognized as a term present in the thesaurus, possibly indicating one of three genes (cf. Figure 3). The disambiguated text fingerprint is shown in Figure 9. Note that the disambiguation algorithm rejected all in-thesaurus senses. Thus, PSA in this text is correctly assumed to have a not-in-thesaurus meaning.

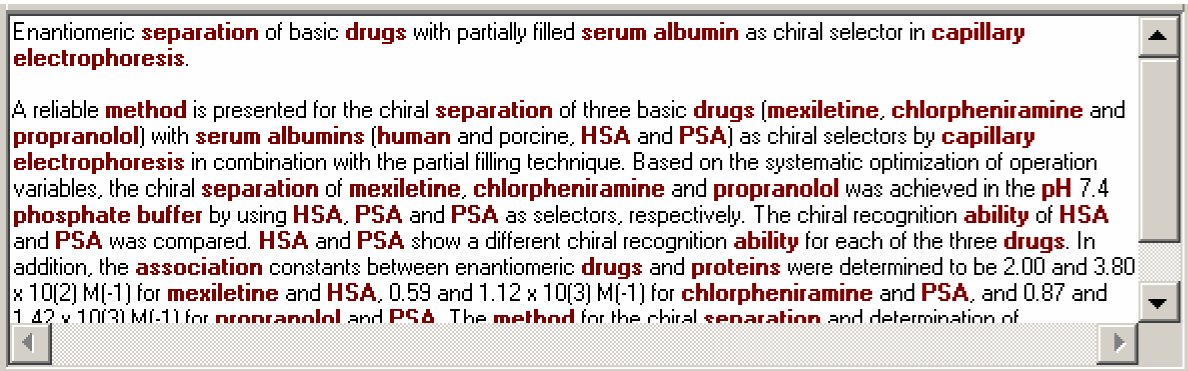

Figure 8. An indexed text which contains the term PSA in a not-in-thesaurus sense.

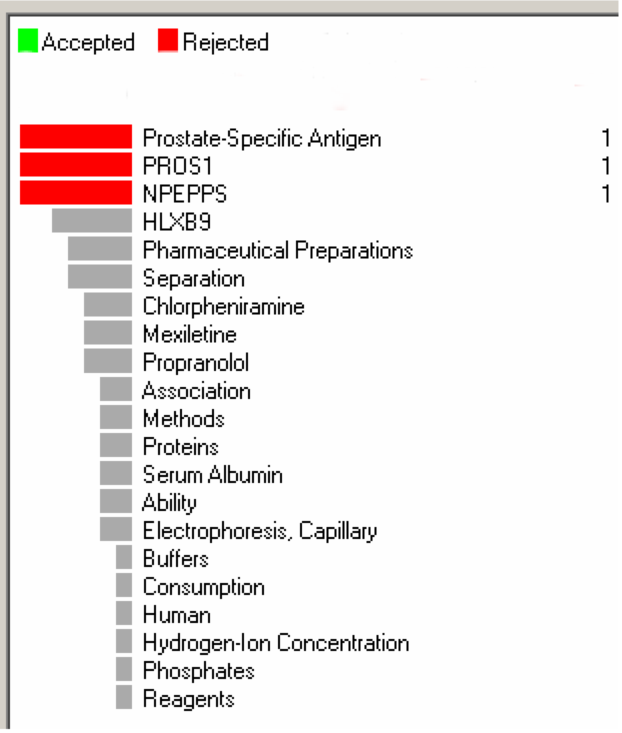

Figure 9. The disambiguated fingerprint corresponding to the text of Figure 8. All in-thesaurus senses of PSA are rejected. The matching scores of the three in-thesaurus senses for this text are: 0.0055 for “Prostate-Specific Antigen”, 0.0055 for “PROS1”, and 0.0042 for “NPEPPS”, all below the not-in-thesaurus thresholds of 0.211, 0.0116, and 0.0052, respectively (thresholds also shown in the reference fingerprints of Figures 4, 5, and 6).
